# Characterization of Gut Microbiota in Prenatal Cold Stress Offspring Rats by 16S rRNA Sequencing

**DOI:** 10.3390/ani10091619

**Published:** 2020-09-10

**Authors:** Jiasan Zheng, Tingting Zhu, Lipeng Wang, Jianfa Wang, Shuai Lian

**Affiliations:** College of Animal Science and Veterinary Medicine, Heilongjiang Bayi Agricultural University, Daqing 163319, China; zjs3399@aliyun.com (J.Z.); ztt895731877@163.com (T.Z.); wlp921947@163.com (L.W.); wjflw@sina.com (J.W.)

**Keywords:** prenatal cold stress, offspring, 16S rRNA, gut microbiota

## Abstract

**Simple Summary:**

Prenatal stress, including prenatal cold stress has long-term effects on offspring’s physical and mental health. Our previous study showed a reduction of anxiety-like behavior in offspring rats suffered from prenatal cold stress. It is well-known that gut microbiota was involved in a variety of physiological activities, such as emotion, cognition, and behavior. However, information on the comparison between prenatal cold stress and gut microbiota in offspring is limited. The current study compared the gut microbiota composition of the prenatal cold stress and non-stress offspring rats. Cold stressed during gestation period showed to change the offspring gut microbiota composition, and Bacteroides and Lactobacillus were significantly increased in prenatal cold stress offspring rat guts. With the hope, cold stress-induced negative effects of animals can be prevented by microbiological interventions.

**Abstract:**

Our previous study showed a reduction of anxiety-like behavior in offspring rats suffered from prenatal cold stress; whether this was related to changes in the offspring gut microbiota is unclear. To obtain the evidence for the role of the gut microbiota in prenatal cold stress offspring, 16S rRNA sequencing technology was used. Male and female offspring rat feces were collected from a room temperature group and a prenatal cold stress group (*n* ≥ 8) for microbial DNA extraction, followed by 16S rRNA sequencing. The results indicated that prenatal cold stress could change the offspring’s gut microbiota composition. Prenatal cold stress significantly upregulates *Lactobacillus*, *Lactobacillus_gasseri*, *Bacteroides*, and *Bacteroides-acidifaciens* in female offspring, whereas prenatal cold stress significantly reduced *Lachnospiraceae* and *Prevotellaceae* in male offspring. These data showed the characterization of gut microbiota in prenatal cold stress offspring rats, and these data suggest that microbiological intervention in the future can potentially prevent the negative effects caused by cold stress to animals.

## 1. Introduction

Many common and complex diseases could be traced back to the very beginning of life. The development of animals is a plastic process, from genotypic to phenotypic development, depending on the environment. The developing fetus responds to internal and external environmental conditions during the sensitive period of cell proliferation, differentiation, and maturation. These lead to changes in the functions of cells, tissues, and organs. In turn, these changes may have short-term or long-term consequences for health and disease susceptibility, either independently or through subsequent interactions between developmental processes and the environment [1,2,3].

Prenatal stress affects the hypothalamic-pituitary-adrenal axis (HPA) of offspring [4], the brain neurotransmitter system, sympathetic nervous system (SNS), and cognitive ability. It also alters the neuromodulation of immune function. Environmental factors may have a long-term effect on offspring physical and mental health [5], and nervous system; for example, prenatal cold stress, which can induce several diseases, causes offspring hypertension [6]. Prenatal stress caused brain development disorders was well established in rodents, associated with anxiety, depression, and other abnormal behaviors in offspring [7]. The offspring that experience prenatal psychosocial stress were at elevated risk of anxiety disorders [8]. Prenatal restraint stress caused long-term behavioral deficits in offspring through microbe and C-C motif chemokine ligand 2-dependent mechanisms [9].

Most of the above studies have confirmed that prenatal stress leads to increased anxiety-like behaviors in offspring. However, the results of the open-field test (OFT) and elevated plus-maze test (EPMT) in our previous studies showed that prenatal cold stress-induced the anxiety-like behavior of the offspring rats reduced [5,10]. It is now believed that gut microbiota was essential for early development and regulation of host physiology, central nervous system functions (such as cognitive function), and the neuroendocrine system (such as HPA axis) [11,12]. An increasing number of studies showed that stress could alter the gut microbiota [13,14], which in turn can influence behavior [15], and affect the biological and behavioral responses of the brain, including anxiety-like and depression-like behaviors. In addition, the gut microbiota is sensitive to temperature stress. Studies have shown that cold stress alters the gut microbiota in mice. Intestinal microorganisms transplanted from a cold exposed environment can increase sensitivity to insulin and increase intestinal size and absorption capacity [16].

Hence, we hypothesized that prenatal cold stress might regulate the behavior by altering the gut microbiota composition of the offspring. Because of the 16S rRNA gene sequencing could obtain the information on any microbial alterations present in the gut of animals, this study aims to find the gut microbiota characterization of prenatal cold stress diminished the anxiety-like behavior by 16S rRNA gene sequencing of the offspring fecal samples.

## 2. Materials and Methods

Procedures involving animals were approved by the Animal Care Committee of the Heilongjiang Bayi Agricultural University (Daqing, China). The experimental protocol was performed by the College of Animal Science and Veterinary Medicine, Heilongjiang Bayi Agricultural University (NO. BYAU20190213).

### 2.1. Experimental Animals

Twenty male (280 ± 20 g) and thirty female (230 ± 20 g, 9–10 weeks of age) SPF Wistar rats were purchased from Changsheng Co. Ltd. (Changchun, China).(the Wistar rats were sensitive to various nutrients and environment temperature, suitable for the study of various nutritional and stress) Management of animals feeding were referred to our previous studies [5,10]. The rats were kept in an artificial intelligence climate chamber for at least seven days to acclimate.

### 2.2. Prenatal Stress

A vaginal smear was taken to determine the proestrus of the female rats. On the day of proestrus, male and female rats mated in a cage in a ratio of 1 to 2 (one male and two female rats were placed in one cage). It was considered gestational day 0 when sperm was observed under the microscope (The males were removed from the cage when pregnancy was detected, and the specific operations are described in our previous studies [10,17]). On gestational day 14, the cold stress group of pregnant rats was placed in a 4 °C artificial intelligence climate chamber; the control group was continued to be kept at the temperature as 22 ± 2 °C. After parturition, the cold stressed groups were transferred to the room temperature climate chamber.

**Figure 1:**
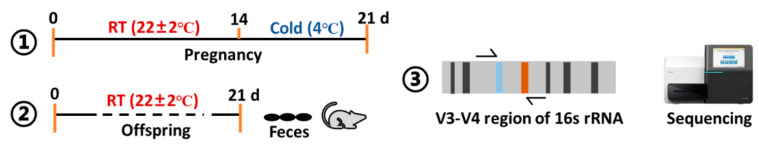
Treatment protocol and schematic workflow to examine the gut microbiota composition.

Twenty-one days after parturition, the offspring were divided into a cold female (CF), cold male (CM), room temperature female (RTF), and room temperature male (RTM) (*n* ≥ 8 in each group). The feces of each group were collected, frozen in liquid nitrogen to solidify (Solidify the bacteria in the sample so that the species and abundance of the sample do not change), then stored at −80 °C until the DNA was extracted for microbiome analysis. The timeline of the treatment protocol was shown in Figure 1.

### 2.3. Microbial Sequencing-16s rRNA

DNA was extracted from feces using a QIA amp DNA Kit (Qiagen). After purity and concentration were measured, the DNA was diluted to 1 ng/μL with sterile water. The PCR was performed with diluted template DNA using region-specific primers (F:5′-CCTAYGGGRBGCASCAG-3′, R:5′-GGACTACH VGGGTWTCTAAT-3′). PCR products were detected by electrophoresis, then purified with Gel Extraction Kit. Amplicon libraries were established using Ion Plus Fragment Library Kit. After Qubit quantification (Qubit dsDNA BR assay, Thermo Fisher, MA, USA) and library detection, the Ion S5TMXL (Thermo Fisher, MA, USA) was used for sequencing.

### 2.4. Microbiome Data Processing

Low-quality parts were sheared from reads by Cutadapt V1.91 (http://cutadapt.readthedocs.io/en/stable/) first. The Raw Reads were obtained by preliminary quality control of truncated Barcode and the primer sequence. The sequences of Read were compared with the species annotation database to obtain Clean Reads (Table 1).

To analyze the diversity of species composition of the samples, Uparse v7.0.1001 [18] was used for clustering for Clean Reads. The sequence cluster was called an Operational Taxonomic Unit (OTU) with 97% identity. Meanwhile, the representative sequences of OTU were selected. On the basis of its algorithmic theory, the sequence with the highest frequency in the OTUs were selected as the representative OTU sequence. Species annotation analysis was carried out by the Mothur method, and the SILVA SSUrRNA database [19] was used to obtain taxonomic information on each taxonomic level. Then, MUSCLE 3.8.31 software was used for multiple sequence alignment, and the system relations of all OTU representative sequences were obtained. Finally, the data of each sample were homogenized. Subsequent Alpha and Beta diversity analyses were based on homogenized data.

Alpha Diversity was used to analyze the microbial community diversity of the within-community. The dilution curve, rank abundance curve, and species accumulation curve were plotted using R software. The differences between groups of Alpha Diversity indices were also analyzed using the above software.

Beta Diversity was a comparative analysis of the microbial community composition of different samples. Principal Co-ordinates Analysis (PCoA) and Non-Metric Multi-Dimensional Scaling (NMDS) diagrams were drawn using R software. Differences in Beta diversity indices between groups were calculated by parametric and non-parametric tests using R software.

### 2.5. Statistical Analysis

The Alpha Diversity and Beta Diversity indices differences were analyzed by parametric and non-parametric tests. A *t*-test and Wilcoxon test were used for two groups. The Tukey test and Wilcoxon test were used for more than two groups. Correlation analysis was performed with the R software, *P* < 0.05 were considered statistically significant.

## 3. Results

### 3.1. OTU Cluster Abundance Analysis

**Figure 2:**
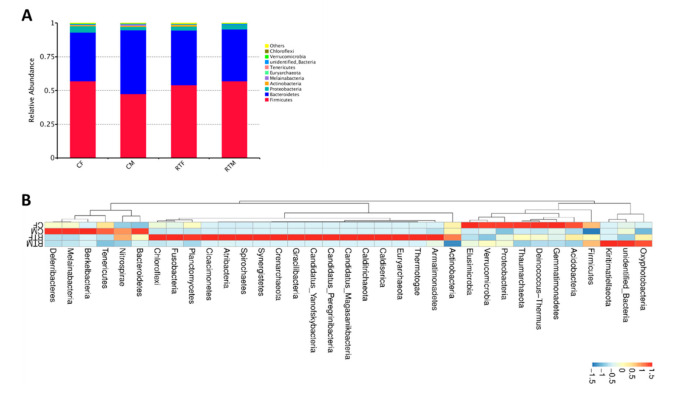
The proportion of dominant phyla in each group. (**A**) Relative abundance histogram of the gut microbiota of the offspring, (**B**) cluster heat map of the gut microbiota in the phylum level. Cold female (CF), cold male (CM), room temperature female (RTF), and room temperature male (RTM).

At the phylum level, Firmicutes, Bacteroidetes, Proteobacteria, Actinbacteria, Melainabacteria, Euryarchaeota, Tenericutes, Unidentified-Bacteria, Verrucomicrobia, and Chloroflexi were the top ten phyla in the offsprings’ gut microbiota. The dominant phyla were Firmicutes, Bacteroidetes, and Proteobacteria, accounting for more than 97% of the phyla. The proportion of dominant phyla in each group is shown in Table 2 and Figure 2A,B.

**Figure 3:**
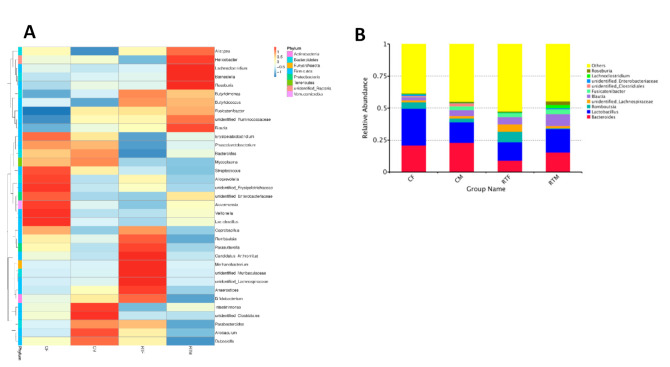
The top ten genera proportions in each group. (**A**) Cluster heat map of the gut microbiota at the genus level, (**B**) relative abundance histogram of the gut microbiota at the genus level. Cold female (CF), cold male (CM), room temperature female (RTF), and room temperature male (RTM).

In the genus level, the top ten genera with the highest abundance were Bacteroides, Lactobacillus, Romboutsia, Unidentified-Lachnospiraceae, Blautia, Fusicatenibacter, Unidentified-Clostridiales, Lachnoclostridium, and Roseburia. The proportions in each group are shown in Table 3 and Figure 3A,B.

### 3.2. Alpha Diversity Analysis

**Figure 4:**
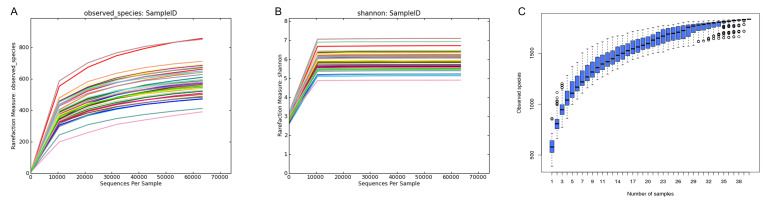
The rarefaction curve and Shannon curve. (**A**) Rarefaction curve of the offspring’s gut microbiota, (**B**) Shannon curve of offspring gut microbiota, (**C**) species cumulative box plot of offspring gut microbiota.

In order to verify whether these data fully reflected the species diversity of gut microbiota, we used the rarefaction curve to analyze each sample. The rarefaction curve and Shannon curve tended to be stable and straight as the sequencing deepened (Figure 4A,B). This indicated that the intestinal microbial diversity of the offspring had been fully detected. As shown in the species cumulative box plot (Figure 4C), with an increase in the number of test samples, the curves gradually became flat. This indicated that the number of samples in this experiment had reached the basic standard level of detection and sufficient to fully reflect the species richness.

### 3.3. Beta Diversity Analysis

**Figure 5:**
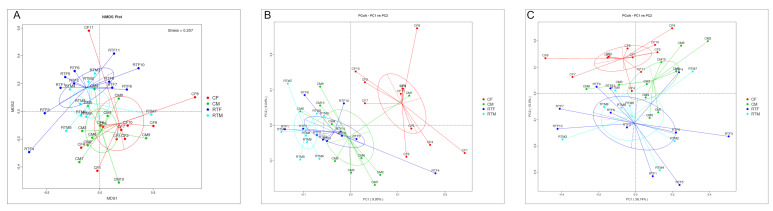
(**A**) Non-Metric Multi-Dimensional Scaling (NMDS) analysis of the gut microbiota of the offspring, (**B**) PcoA analysis of the gut microbiota of the offspring (unweighted), (**C**) Principal Co-ordinates Analysis (PcoA) analysis of the gut microbiota of the offspring (weighted). Cold female (CF), cold male (CM), room temperature female (RTF), and room temperature male (RTM).

In order to analyze the influence of prenatal cold stress on the gut microbiota structure of offspring rats, we selected Non-Metric Multi-Dimensional Scaling (NMDS) and Principal Co-ordinates Analysis (PCoA) for intuitive analyses. As shown in Figure 5A, the confidence ellipses of group RTM and CM have been separated for the most part, and the confidence ellipses of RTF and CF have been completely separated; this indicated that the structure of gut microbiota in offspring rats had changed significantly, due to prenatal cold stress. However, the confidence ellipses of RTM and RTF offspring rats partially overlapped, indicating, thereby that the gut microbiota structure of offspring rats had sex differences. As shown in Figure 5B,C, the confidence ellipses of CM and CF have been completely separated; this also indicated that the influence of prenatal cold stress on the intestinal microflora of the offspring differed in male and female rats.

Multi Response Permutation Procedure (MRPP) was used to verify the prenatal cold stress effect on the gut microbiota structure of offspring rats. The results were consistent with the PCoA and NMDS analysis, A > 0 and P < 0.05 (Table 4). This indicated that prenatal cold stress had a significant effect on the gut microbiota structure of offspring rats.

### 3.4. Screening of Microorganisms in Response to Prenatal Cold Stress

**Figure 6:**
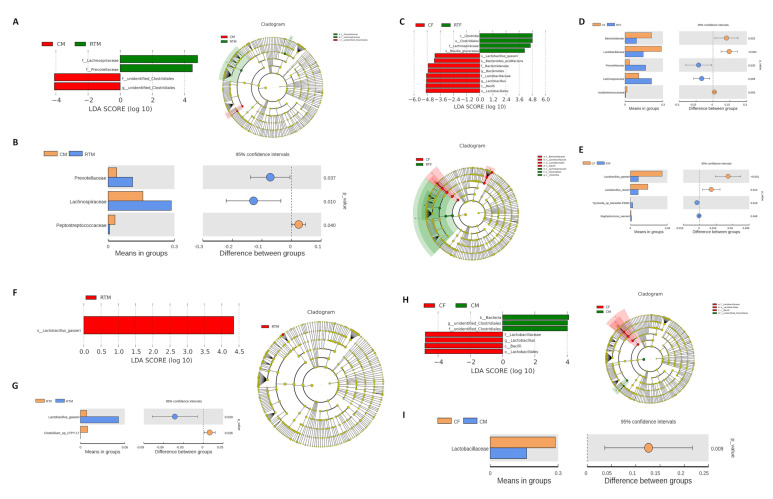
(**A**) LDA (Linear discriminant analysis) value distribution histogram between CM and RTM groups, (**B**) *t*-test analysis between CM-RTM groups, (**C**) LDA value distribution histogram between CF and RTF groups, (**D**) T-test analysis between CF-RTF groups (genus level), (**E**) *t*-test analysis between CF-RTF groups (species level), (**F**) LDA value distribution histogram between RTM and RTF groups, (**G**) *t*-test analysis between RTM-RTF groups, (**H**) LDA value distribution histogram between CF and CM groups, (**I**) *t*-test analysis between CF-CM groups. Cold female (CF), cold male (CM), room temperature female (RTF), and room temperature male (RTM).

LDA Effect Size (LefSe) and *t*-test were used to identify the key microorganisms that showed the strongest response to prenatal cold stress. Compared to the RTM group, Lachnospiraceae and Prevotellaceae were significantly decreased in the CM group (P < 0.05, LDA = 4) (Figure 6A,B); compared to the RTF group, *Bacilli*, *Lactobacillales*, *Lactobacillaceae*, *Lactobacillus*, *Bacteroides*, *Bacteroidaceae*, and *Lactobacillus_gasseri* were significantly increased in the CF group (P < 0.05, LDA = 4) (Figure 6C–E); compared to the RTM group, *Lactobacillus_gasseri* was significantly more in the RTF group (Figure 6F,G); compared to the CF group, *Bacilli*, *Lactobacillales*, *Lactobacillaceae*, and *Lactobacillus* were significantly increased in the CM group (Figure 6H,I).

## 4. Discussion

In our present study, the intestinal microbial diversity of the offspring had been fully detected, and alpha diversity analysis showed species richness of the offspring’s gut microbiota. Beta diversity analysis showed the structure of gut microbiota had changed significantly, due to prenatal cold stress. We found that *Bacteroides* in the fecal samples of the CF group was more abundant than in the RTF group. One study demonstrated that the oral treatment of maternal immune activation (MIA) mouse offspring with human commensal *Bacteroides* fragilis can correct gut permeability, alter the microbial composition, and ameliorate defects in communicative, stereotypic, anxiety-like, and sensorimotor behaviors [20]. Prenatal cold stress may reduce the offspring’s anxiety-like behavior by increasing the proportion of Bacteroidetes in the offspring’s gut.

Moreover, we also found that prenatal cold stress caused an increase in the proportion of *Lactobacillus* in the offspring’s gut. *Lactobacillus* is present in probiotics, which can regulate the response to stress and anxiety symptoms [21,22,23]. It has been demonstrated that feeding *Lactobacillus rhamnosus* increased GABA (γ-aminobutyric acid) receptors in the hippocampus, and reduced stress-induced anxiety and depression-related behavior [24]. GABAergic neurotransmission in the hippocampus makes a decisive difference in the modulation of behavior and memory processes [25,26]. GABA-B receptors are related to mood and anxiety disorders, and blocking these receptors could reduce anxiety [27]. In agreement, in our previous study, we also found prenatal cold stress inhibited the expression of GABA-B2 receptors in the offspring rat’s hippocampus. These results imply that the increase in *Lactobacillus* induced by prenatal cold stress, promotes the expression of GABA receptors in the hippocampus of the offspring, thereby reducing the offspring’s anxiety-like behavior. In addition, the gut microbiota affects nervous system functions in the host [28,29]. *Bacteroides* and *Lactobacillus* produce large amounts of GABA [30,31]. Prenatal cold stress-induced an increase in *Bacteroides* and *Lactobacillus* in the offspring’s gut; hence, we speculated that prenatal cold stress also increased GABA levels in the offspring.

In this study, we observed that the fecundity of *Lactobacillus_gallinis* in cold-stressed offspring was significantly higher than that in the room temperature group. It has been reported that *Lactobacillus gallinis* can produce a variety of bacteriocins, and can inhibit harmful bacteri. It is also involved in promoting intestinal stability and maintaining vaginal health, preventing allergic reactions, and inhibiting *Helicobacter pylori*. Prenatal cold stress-induced probiotic elevation may be regulated by the negative feedback mechanism of the organism.

Excitement and inhibition in healthy animals are two basic neural processes that are opposed to each other. A moderate amount of anxiety could help energize and mobilize individual energy to deal with stress, so increasing the chances of individual survival and continuation. When this anxiety was suppressed, the ability to respond to stress was also reduced. From our previous behavioral test results, prenatal cold stress could reduce the offspring’s ability to react to stress. Moreover, the negative effects caused by cold stress to animals can be prevented by microbiological interventions.

## 5. Conclusions

In conclusion, *Bacteroides* and *Lactobacillus* were significantly increased in prenatal cold stress the offspring rats’ gut. These data showed the characterization of gut microbiota in prenatal cold stress offspring rats, with the hope that cold stress-induced negative effects of animals can be prevented by microbiological interventions.

## Figures and Tables

**Table 1:** Data preprocessing statistics and quality control information.

Sample	Raw_Reads	Clean_Reads	Base(nt)	AvgLen(nt)	Q20	GC%	Effective%
CF1	92,260	89,261	22,514,767	252	82.14	52.13	96.75
CF2	83,650	80,149	20,243,525	252	83.8	51.94	95.81
CF3	83,298	80,401	20,316,690	252	82.54	52.07	96.52
CF4	83,297	80,125	20,214,254	252	83.66	52.48	96.19
CF5	85,330	80,021	20,190,759	252	83.37	51.75	93.78
CF6	85,139	80,086	20,229,884	252	83.64	52.26	94.06
CF7	84,444	80,156	20,262,129	252	82.6	52.91	94.92
CF8	93,369	90,450	22,854,929	252	83.14	52.95	96.87
CF9	82,300	80,012	20,193,261	252	83.36	49.78	97.22
CF10	83,407	80,210	20,227,906	252	82.39	51.75	96.17
CF11	77,267	74,592	18,829,804	252	88.38	51.82	96.54
CM1	85,000	80,135	20,229,046	252	84.22	52.48	94.28
CM2	82,853	80,271	20,229,164	252	83.8	50.55	96.88
CM3	85,402	80,218	20,248,437	252	87.64	52.1	93.93
CM4	82,193	80,067	20,203,569	252	89.1	53.63	97.41
CM5	85,686	80,027	20,196,156	252	82.52	53	93.4
CM6	85,413	80,192	20,234,272	252	81.6	51.65	93.89
CM7	83,505	80,110	20,252,100	252	80.29	51.82	95.93
CM8	82,851	80,161	20,257,659	252	82.13	52.09	96.75
CM9	83,032	80,250	20,225,862	252	85.33	50.51	96.65
CM10	94,549	91,345	23,051,906	252	86.17	51.74	96.61
RTF1	89,793	85,797	21,722,030	253	85.33	52.34	95.55
RTF2	84,096	80,166	20,241,950	252	87.34	53.06	95.33
RTF3	85,131	80,209	20,322,631	253	81.71	52.24	94.22
RTF4	82,078	80,181	20,237,808	252	82.2	51.82	97.69
RTF5	103,541	99,559	25,148,508	252	88.31	52.24	96.15
RTF6	85,361	80,235	20,254,767	252	88.53	51.95	93.99
RTF7	71,314	68,814	17,387,481	252	78.71	53.17	96.49
RTF8	84,943	80,114	20,195,893	252	83.01	52.6	94.32
RTF9	84,557	80,148	20,248,562	252	83.97	52.43	94.79
RTF10	82,732	80,286	20,269,324	252	84.38	53.05	97.04
RTF11	83,449	80,090	20,217,831	252	88.04	52.86	95.97
RTM2	83,144	80,084	20,225,142	252	84.92	51.63	96.32
RTM3	83,169	80,105	20,284,526	253	81.31	52.78	96.32
RTM4	83,560	80,200	20,254,101	252	87.61	52.47	95.98
RTM5	85,973	79,380	20,034,819	252	85.79	52.68	92.33
RTM6	83,612	80,295	20,278,888	252	81.36	52.74	96.03
RTM7	82,830	80,147	20,222,220	252	84.19	50.56	96.76
RTM8	84,339	80,215	20,236,766	252	88.18	52.92	95.11
RTM9	83,915	80,047	20,206,904	252	81.54	52.35	95.39

**Table 2:** The proportion of dominant phylum in each group.

Groups	Firmicutes	Bacteroidetes	Proteobacteria
**RYM**	57.02%	38.04%	3.18%
**CM**	47.57%	47.28%	2.48%
**RTF**	54.11%	40.40%	3.06%
**CF**	57.00%	36.17%	4.57%

**Table 3:** The top ten bacteria with the highest abundance at the genus level.

Groups	Bacte	Lacto	Romb	Un_-_Lachn	Blaua	Fusica	Un_-_Clostri	Un_-_Entero	Lachn	Roseb
**RYM**	15.51	18.28	0.67	1.54	9.38	3.78	0.04	0.74	2.53	2.94
**CM**	22.93	16.11	3.09	1.72	4.76	2.96	2.34	0.13	0.25	0.87
**RTF**	9.03	14.39	8.20	5.84	5.52	3.24	0.12	0.08	0.41	0.76
**CF**	20.97	28.75	4.99	1.52	2.40	0.31	0.59	1.21	0.51	0.37

**Table 4:** MRPP analysis.

Group	A	Observed-Delta	Expected-Delta	Significance
CF-RTF	0.05108	0.6782	0.7147	0.001
RTF-RTM	0.02732	0.683	0.7022	0.035
CF-RTM	0.04646	0.652	0.6837	0.001
CM-RTF	0.04115	0.6967	0.7266	0.004

Cold female (CF), cold male (CM), room temperature female (RTF), room temperature male (RTM).
